# Ovarian Tuberculosis masquerading as ovarian cancer in HIV infected patient: a plea to avoid unnecessary surgery

**DOI:** 10.11604/pamj.2014.19.210.5072

**Published:** 2014-10-27

**Authors:** Ballah Akawu Denue, Salisu Aliyu Kwayabura, Haruna Asura Ngadda

**Affiliations:** 1 Department of Medicine, College of Medical Sciences, University of Maiduguri, PMB 1069, Maiduguri, Nigeria; 2 Department of Obstetrics and Gynaecology, College of Medical Sciences, University of Maiduguri, PMB 1069, Maiduguri, Nigeria; 3 Department of Histopathology, College of Medical Sciences, University of Maiduguri, PMB 1069, Maiduguri, Nigeria

**Keywords:** Ovarian TB, ovarian cancer, HIV-TB co infection

## Abstract

Female patients who present with adnexial mass and weight loss should not be presumed to have ovarian carcinoma until after extensive investigation. This is to avoid the mistake of radical surgery with its attendant morbidity and mortality. An important disease to consider in our environment is ovarian TB that respond well to medication. A 35 year old HIV-1 positive house wife presented with fever, persistent vomiting, progressive weight loss, vague abdominal pain and swelling. Patient occasionally ingest unpasteurized milk since childhood but had no sustained contact with adult with chronic cough. She had no menstrual abnormality. Imaging studies revealed right ovarian mass measuring 11.8cmx10cm. Right ovarian malignancy was highly suspected, for which she underwent exploratory laporotomy. Histopathology result was consistent with tuberculous granuloma. Chest radiograph was normal. Her CD4 count was 541cells/ul. Patient was commenced on anti tuberculotic therapy based on the Nigerian National TB control and she responded well. Tuberculosis of the ovary can masquerade as ovarian cancer, especially among HIV patients in regions where TB-HIV co infections is endemic, it should be ruled out before performing extended surgery.

## Introduction

Tuberculosis remains a major public health concern especially in third world countries. Although the incidence of TB cannot be measured directly, it is estimated that it has a devastating impact in Sub Saharan Africa and Asia with 13 countries mostly from this region accounting for nearly 75% of global TB cases [1]. HIV epidemic has altered TB dynamic and has lead to its resurgence in communities that bear the burden of the two diseases [2]. TB present as pulmonary (PTB) or extra pulmonary (EPTB). Genital TB (GTB) a form of EPTB is rare in western societies based on paucity of data but not uncommon in areas where HIV-TB co infection is common such as developing nations [3, 4]. Quantitative and qualitative defect in cellular immunity predisposes patients to gynaecological malignancies and infections including TB [5]. Female patients presenting with pelvic masses and ascites are often mistaken for malignant ovarian tumours and can pose a diagnostic and therapeutic dilemma especially in regions where there are dearth of specialist and facilities for evaluation in the face of increasing incidence of TB infection [6]. In this report, we present a rare case of unilateral ovarian TB misdiagnosed as ovarian cancer in a woman within a reproductive group that had a explotory laporotomy at a secondary health facility.

## Patient and observation

**Figure 1:**
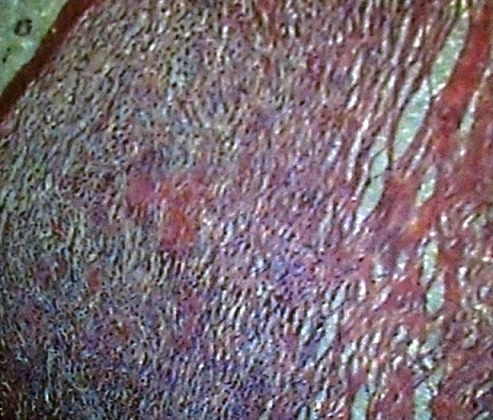
Photomicrograph showing caseating granuloma consistent with tuberculosis (H/E X164)

Mrs F U, a 35 year old house wife presented with 3 weeks remittent fever, persistent vomiting, progressive weight loss, vague abdominal pain and swelling that defied medications including antimalarials, antibiotics and antiemetics at a pheripheral hospital. Patient occasionally ingest unpasteurized milk since childhood but had no sustained contact with adult with chronic cough. Patient had abdominal ultrasound and other blood investigations on request by medical officer, abdomino-pelvic scan done revealed right ovarian mass measuring 11.8cm x 10cm displacing the uterus anteriorly. Diagnosis of right ovarian cancer was made for which the patient had explotory laporotomy. Histology of the excised lesion showed tuberculous granuloma, on this account the patient was referred to our hospital; a tertiary health facility and center of excellence in infectious diseases and immunology for further evaluation. On clinical examination the patient was wasted, pale, anicteric and had no peripheral lymph node enlargement. Abdomen was flat with healing paramedian incision from xyphiternum to pubic symphysis, with no organomegally or ascites. Other systemic examination was unremarkable. She had haemoglobin (Hb) of 10.3g/dl, features of iron deficiency anaemia on blood film, WBC of 13.8 x109/l, Differentials (lymphocyte 65%, Neutrophil 30%), ESR 110mm/hr. Liver function test and renal function test including urinalysis were all within normal limit. No abnormality was detected on chest radiograph. Macroscopically the cut section looked grayish white; the section showed fragments of tissue displaying a caseating granulomatous inflammation composed of large areas of central caseous necrosis surrounded by epitheliod cells, multinucleated gaint cells, lymphocytes and fibroblasts in keeping with tuberculous granuloma as in Figure 1. Patient tested HIV-1 after counseling. Her CD4 count was 541cells/ul. Patient was subsequently commenced on anti tuberculotic therapy based on the Nigerian National TB control adopted from WHO DOTS programme that recommend quadriple therapy of isoniazid, rifampicin, ethambutol and pyrizinamide for the 2 month intensive phase and continued with isoniazid/rifampicin for another 4 months as continuation phase. Patient responded well to medication as evidenced by disappearance of the presenting symptoms and improvement in appetite within three weeks of medication.

## Discussion

Although the incidence of Genital TB among women are difficult to predict with certainty because it may be asymptomatic and undiagnosed, it affect about 12% of women with PTB and 15%-20% women with EPTB. Primary GTB is extremely rare, it almost exclusively reaches the genital tract from a focus elsewhere [7, 8]. Haematogenous spread of the TB bacilli from primary focus such as lungs occurs in almost 90% of cases, other less common means is through the lymphatic system or directly from gastrointestinal tract, mesenteric lymph nodes or peritoneum. Moreover, rarely cases of primary inoculation during sexual intercourse with infected partner with tuberculous lesion genitalia are reported [9]. Unlike fallopian tubes that is affected in about 95% to 100% of GTB, uterus in about 50% to 60% of cases, involvement of the ovaries is rarely seen in only 20% [9, 10]. Our patient is 35 years, this is not surprising as 80% to 90% of GTB cases occur within the ages of 20 and 40 years. Her Gender and HIV sero positive status are risk factors for GTB [9, 10].

Changes in demographic characteristics of tuberculosis cases and HIV epidemic are thought to be responsible for increase in cases of ETB cases including GTB especially in communities where cases of TB-HIV are common [11]. Our patient presented with vague abdominal pain, right adnexial mass and constitutional symptoms. Although we had no opportunity to assay serum CA-125 level, which is not readily available in our environment, use of CA-125 in discriminating metastatic ovarian cancer from other disease conditions is extremely difficult. CA -125 a tumour associated antigen is no specific, as it is elevated in a variety of conditions including TB [12]. We also had no opportunity of assaying Ascitic fluid adenosine deaminase (ADA), Omental biopsy and peritoneal was also not done to rule out involvement of the peritoneum. GTB especially in the setting of HIV infection may be difficult to detect as they can present with myriad of gynaecological malignancies and infections. The diagnosis of GTB is a clinical challenge and is hardly made based on clinical symptoms of its low specificity. Therefore, elaborate evaluation including pelvic ultrasound, X ray, mantoux test, bacteriological cultivation, Ziel-Neelson staining for Acid Fast Bacilli, PCR analysis and less invasive diagnostic laporoscopic procedures should be done instead of hasty radical exploratory laporotomy as was done in this case. Although the CD4 count of this patient was 541 cells/ul, the defect in cellular immunity may be functional since the cyflow machine captures both mature and immature CD4 T cells [13]. This defect in immunity might explain EPTB manifestation without subsequent focus, unlike immunocompetent patients that manifest with lesions of primary and dissiminated TB that can heal or remain focus of dissemination when there is reactivation or re infection [14].

## Conclusion

We report an unusual presentation of a common disease masquerading as cancer of ovary, even though it was interesting to see marked improvement in patients clinical condition on anti tubercular medication within a month, diagnosis of TB involving the right ovary was done after post operatively based on histopathology result. Given the high prevalence of TB especially among HIV positive patients in our environment, Genital TB should be ruled out before performing extended surgery.
